# Supplementary material for: Fibroblast growth factor–inducible 14 regulates satellite cell self-renewal and expansion during skeletal muscle repair
Source: JCI Insight. 2025 Jan 28;10(5):e187825. doi: 10.1172/jci.insight.187825 (PMC11949035; doi:10.1172/jci.insight.187825)
Supplement: Supplemental data [file jciinsight-10-187825-s136.pdf]

Supplemental Data File

Fibroblast growth factor-inducible 14 regulates satellite cell self-renewal and expansion during skeletal muscle repair

By

Meiricris Tomaz da Silva, Aniket S. Joshi, and Ashok Kumar

This file contains Supplemental Figures, 1-5 and Supplemental Table 1-3.

Supplemental Figures' and Legends

Supplemental Figure 1.

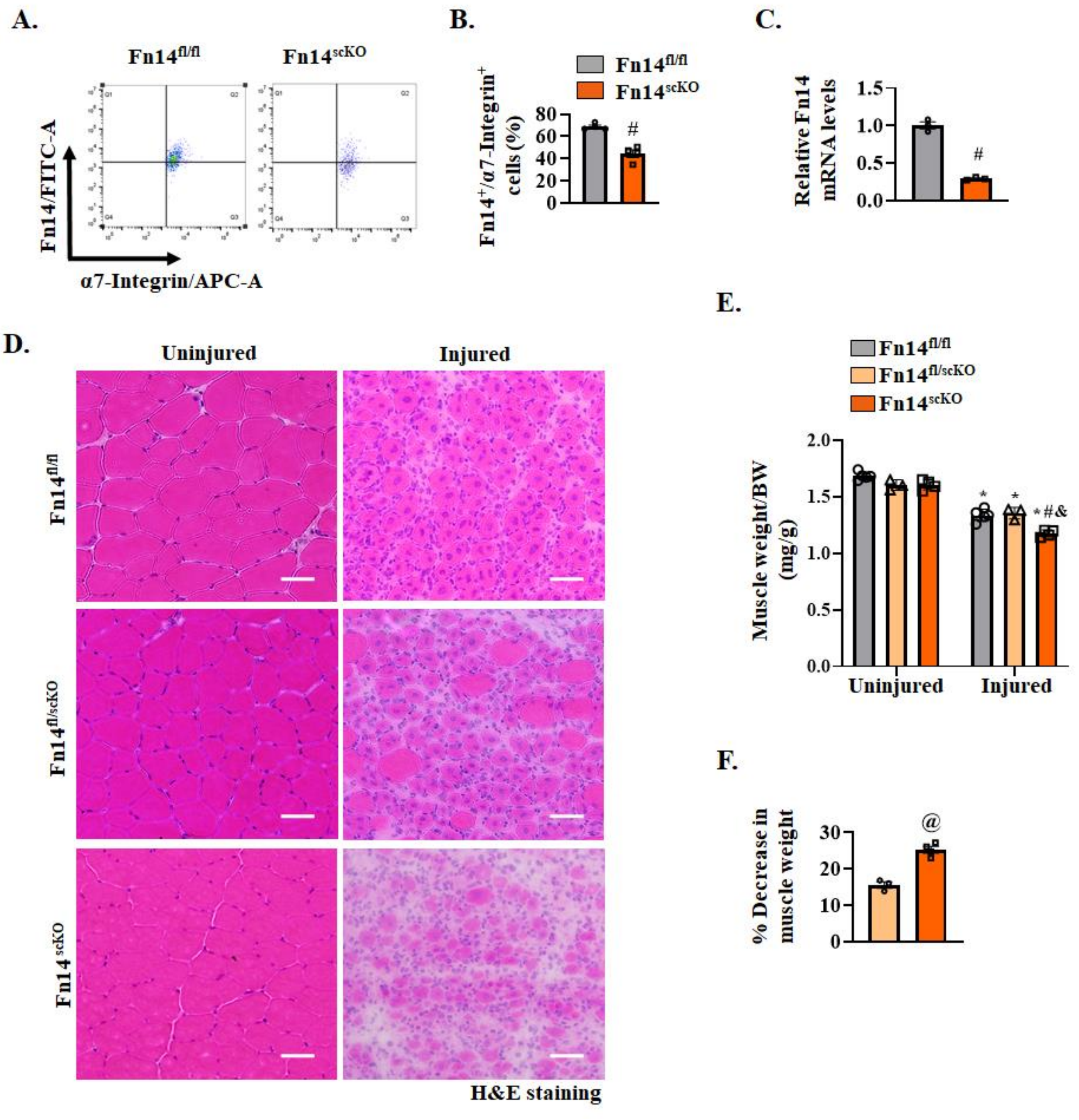

Supplemental FIGURE 1. **Fn14 signaling in satellite cells mediates muscle regeneration.** **(A)** Primary mononuclear cells were isolated from TA muscle of Fn14$^{fl/fl}$ and Fn14^{scKO} mice and subjected to FACS analysis for the expression of α7-integrin and Fn14. Representative scatter plots of FACS-based demonstrating Fn14^{+} and α7-integrin^{+} cells in 5d-injured TA muscle of Fn14$^{fl/fl}$ and Fn14^{scKO} mice. **(B)** Quantification of the percentage of Fn14^{+}/α7-integrin^{+} cells in 5d-injured TA muscle of Fn14$^{fl/fl}$ and Fn14^{scKO} mice assayed by FACS. **(C)** Relative mRNA

levels of Fn14 in isolated satellite cells of 5d-injured TA muscle of $Fn14^{fl/fl}$ and $Fn14^{scKO}$ mice. n=3-4 mice in each group. #$p \leq 0.05$, values significantly different from corresponding 5d-injured TA muscle of $Fn14^{fl/fl}$ mice analyzed by unpaired Student *t* test. **(D)** Representative photomicrographs of H&E stained sections of uninjured and injured TA muscles of $Fn14^{fl/fl}$, $Fn14^{fl/wt}$; Pax7-CreERT2 ($Fn14^{fl/scKO}$) and $Fn14^{scKO}$ mice. Scale bar, 50 μm. **(E)** Uninjured and 5d-injured TA muscle wet weight normalized by body weight (BW) of $Fn14^{fl/scKO}$ and $Fn14^{scKO}$. **(F)** Percentage decrease in wet weight of TA muscle of $Fn14^{fl/scKO}$ and $Fn14^{scKO}$ after injury. n= 3-4 mice in each group. All data are presented as mean ± SEM. *$p \leq 0.05$, values significantly different from contralateral uninjured muscle of $Fn14^{fl/fl}$ or $Fn14^{fl/scKO}$ mice; $^{\&}p \leq 0.05$, values significantly different from corresponding 5d-injured TA muscle of $Fn14^{fl/fl}$ mice analyzed by two-way ANOVA followed by Tukey's multiple comparison test. $^{@}p \leq 0.05$, values significantly different from corresponding $Fn14^{fl/scKO}$ mice analyzed by unpaired Student *t* test.

Supplemental Figure 2.

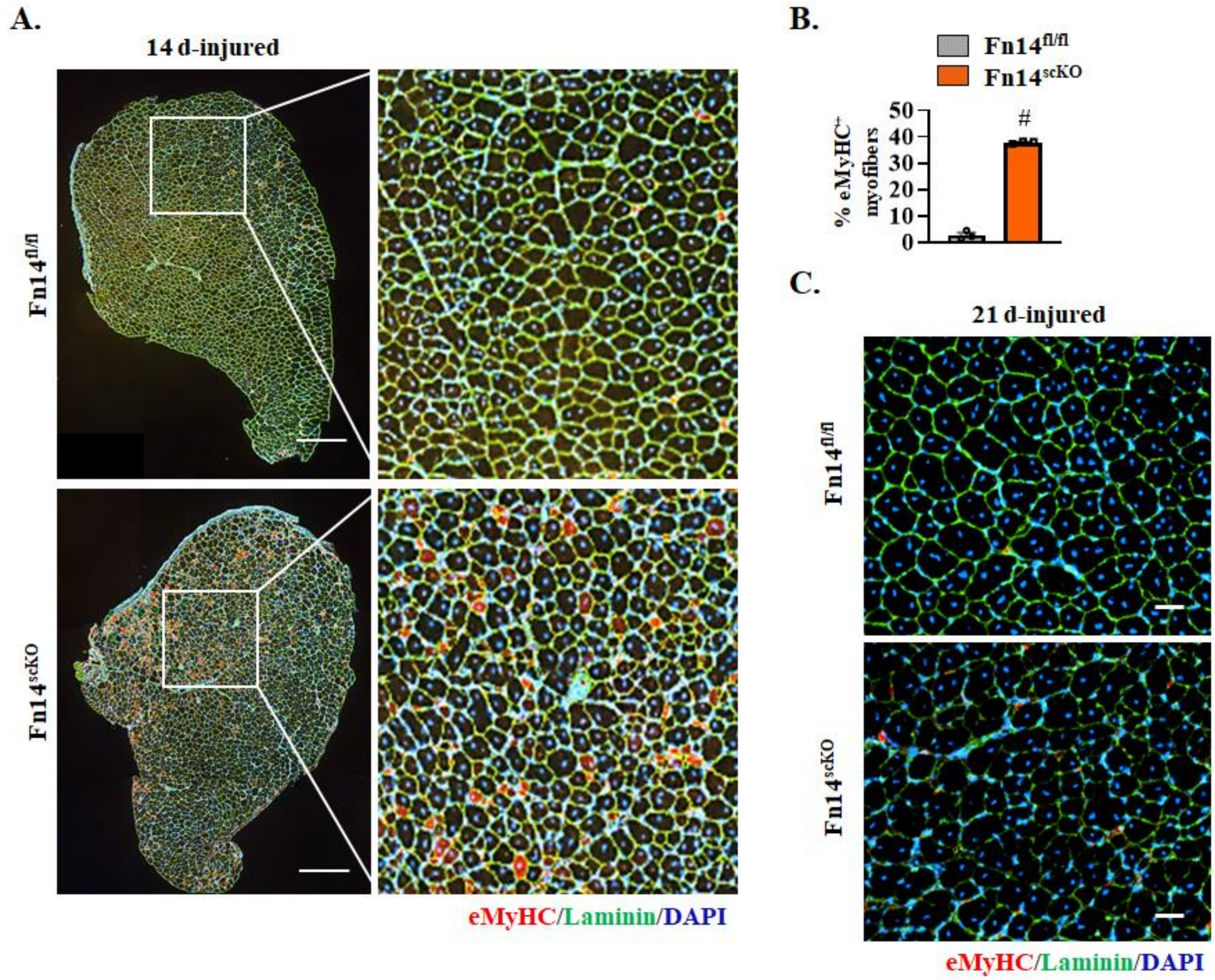

Supplemental FIGURE 2. **Deletion of Fn14 in satellite cells delays muscle regeneration. (A)** Representative photomicrographs of transverse sections of 14d-injured TA muscle of $Fn14^{fl/fl}$ and $Fn14^{scKO}$ mice after immunostaining for eMyHC and laminin protein and DAPI staining. Left panel: whole muscle section; right panel: magnified view of the selected region. Scale bar: 400 µm. **(B)** Quantification of percentage of $eMyHC^{+}$ myofibers in 14d-injured TA muscle section of $Fn14^{fl/fl}$ and $Fn14^{scKO}$ mice. **(C)** Representative photomicrographs of transverse sections of 21d-injured TA muscle of $Fn14^{fl/fl}$ and $Fn14^{scKO}$ mice after immunostaining for eMyHC and laminin protein and DAPI staining. Scale bar: 50 µm. n= 3 mice in each group. Data are presented as mean ± SEM. #$p \leq 0.05$, values significantly different from corresponding muscle of $Fn14^{fl/fl}$ mice analyzed by unpaired Student *t* test.

Supplemental Figure 3.

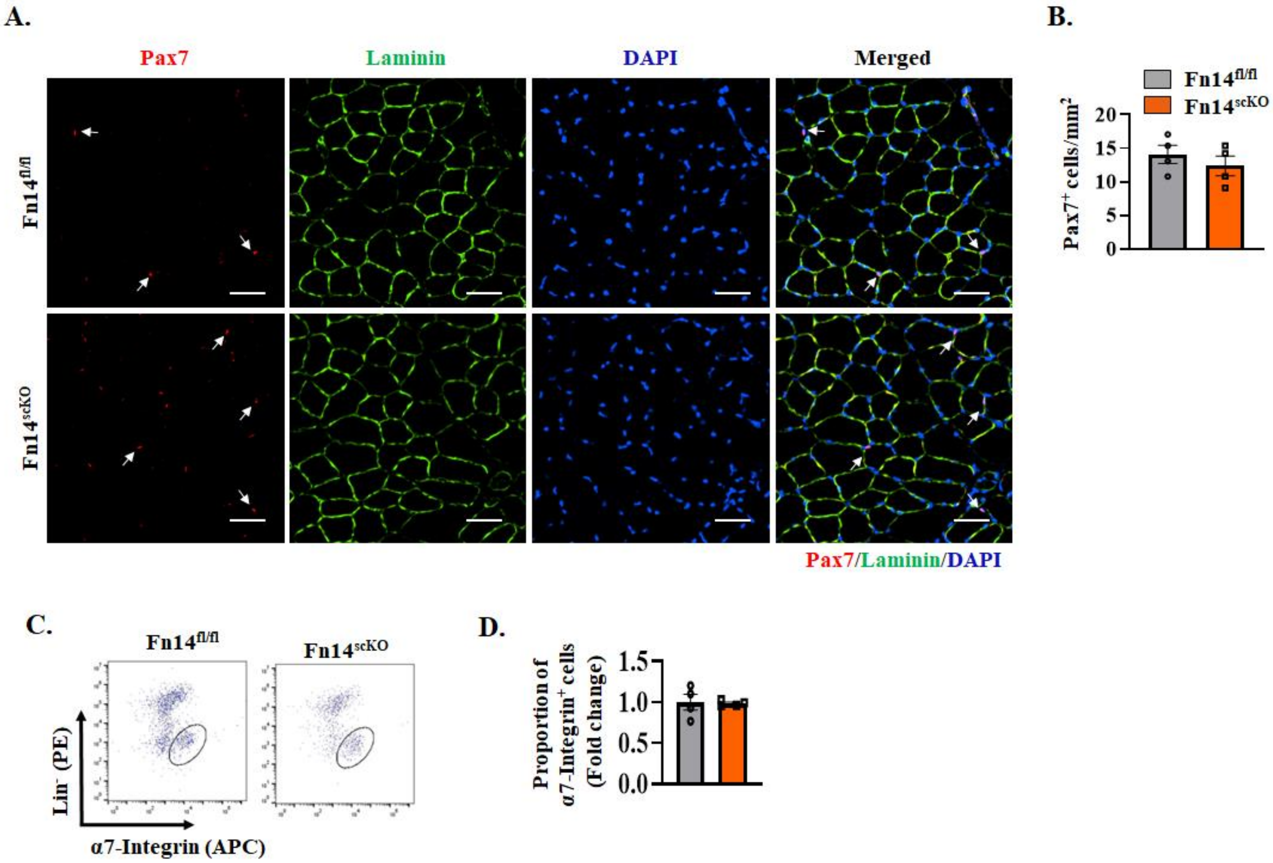

Supplemental FIGURE 3. Targeted deletion of Fn14 does not affect satellite cell number in uninjured muscle. (A) Representative photomicrographs of uninjured TA muscle sections of $Fn14^{fl/fl}$ and $Fn14^{scKO}$ mice after immunostaining for Pax7 (red) and laminin (green) protein. Nuclei were counterstained with DAPI. Scale bar: 50 µm. White arrows point to $Pax7^{+}$ satellite cells. **(B)** Average number of $Pax7^{+}$ cells per unit area in uninjured TA muscle of $Fn14^{fl/fl}$ and $Fn14^{scKO}$ mice. n= 4 mice in each group. **(C)** Single cell suspensions were isolated from TA muscle of $Fn14^{fl/fl}$ and $Fn14^{scKO}$ mice were subjected to FACS analysis for satellite cells. Representative FACS dot plots demonstrating the percentage of α7-integrin^{+} cells in uninjured TA muscle of $Fn14^{fl/fl}$ and $Fn14^{scKO}$ mice. **(D)** Quantification of α7-integrin^{+} satellite cells in uninjured TA muscle of $Fn14^{fl/fl}$ and $Fn14^{scKO}$ mice assayed by FACS. All data are presented as mean ± SEM. No statistically significant difference was obtained between the two genotypes by unpaired Student *t* test.

Supplemental Figure 4.

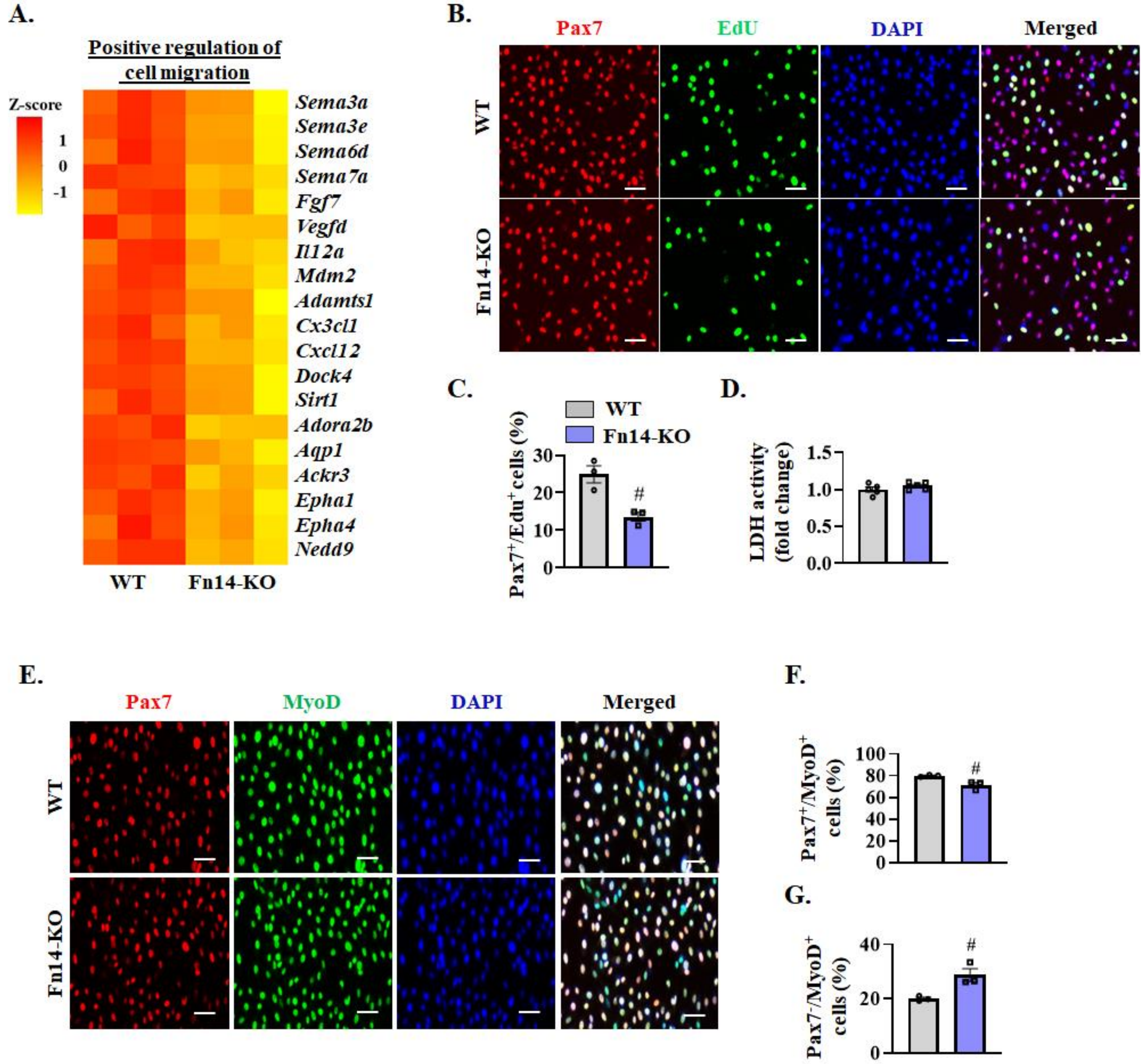

Supplemental FIGURE 4. **Effect of deletion of Fn14 on satellite cell survival and proliferation. (A)** Heatmap representing selected genes associated with the positive regulation of cell migration in WT and Fn14-KO cultures generated by analysis of RNA-seq dataset. **(B)** WT and Fn14-KO myoblasts were seeded at equal number and pulse-labeled with EdU for 60 min followed detection of EdU^+ nuclei and immunostaining for Pax7 protein. Representative photomicrographs of merged images are presented here. Scale bar: 50 µm. **(C)** Quantification of percentage of $Pax7^+/EdU^+$ cells in WT and Fn14-KO cultures. **(D)** Relative amounts of lactate dehydrogenase (LDH) in supernatants of WT and Fn14-KO cultures. n= 3 biological replicates in each group. **(E)** Representative photomicrographs of WT and Fn14-KO primary myoblast cultures after immunostaining for Pax7 and MyoD protein. Nuclei were counterstained by DAPI. Scale bar: 50 µm. Quantification of percentage of **(F)** $Pax7^+/MyoD^+$ (proliferating), and **(G)** $Pax7^-/MyoD^+$ (differentiating) cells in WT and Fn14-KO cultures. n= 3 biological replicates in

each group. All data are presented as mean ± SEM. #$p \leq 0.05$, values significantly different from WT cultures analyzed by unpaired Student *t* test.

Supplemental Figure 5.

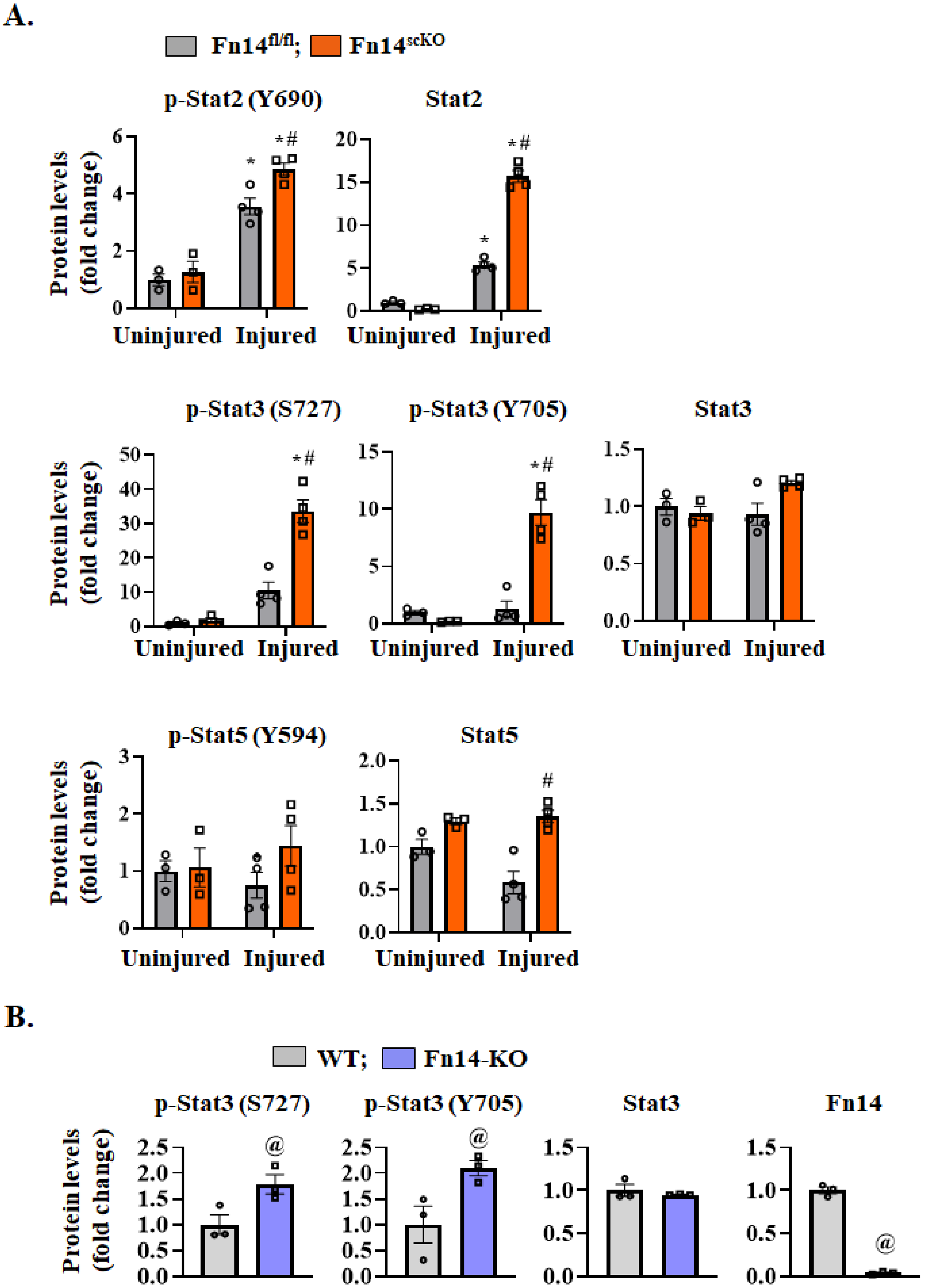

Supplemental FIGURE 5. Fn14 regulates Stat signaling in injured muscle and cultured myoblasts. (A) Densitometry analysis of immunoblots (shown in main Figure 7B) demonstrates fold change in the levels of various phosphorylated and total Stat2, Stat3, and Stat5 protein in uninjured and 5d-injured TA muscle of $Fn14^{fl/fl}$ and $Fn14^{scKO}$ mice. n= 3-4 mice in each group.

(B) Fold change in the levels of phosphorylated and total Stat3 protein in WT and Fn14-KO cultured myoblasts. n= 3 biological replicates in each group. All data are presented as mean ± SEM. *p ≤ 0.05, values significantly different from contralateral uninjured muscle of $Fn14^{fl/fl}$ or $Fn14^{scKO}$ mice; #p ≤ 0.05, values significantly different from corresponding injured TA muscle of $Fn14^{fl/fl}$ mice analyzed by two-way ANOVA followed by Tukey's multiple comparison test. $^{@}$p ≤ 0.05, values significantly different from WT cultures analyzed by unpaired Student *t* test.

Supplemental Table 1. List of primers used for PCR/qRT-PCR analysis.

**Name**	**Forward primer (5'-3')**	**Reverse primer (5'-3')**
Fn14	AAGTGCATGGACTGCGCTTCTT	GGAAACTAGAAACCAGCGCCAA
Pax-7	CAGTGTGCCATCTACCCATGCTTA	GGTGCTTGGTTCAAATTGAGCC
Myod1	TGGGATATGGAGCTTCTATCGC	GGTGAGTCGAAACACGGATCAT
Myh3	ACATCTCTATGCCACCTTCGCTAC	GGGTCTTGGTTTCGTTGGGTAT
Myog	CATCCAGTACATTGAGCGCCTA	GAGCAAATGATCTCCTGGGTTG
Notch1	CAGGAAAGAGGGCATCAG	AGCGTTAGGCAGAGCAAG
Notch2	GCAGGAGCAGGAGGTGATAG	GCGTTTCTTGGACTCTCCAG
Notch3	GTCCAGAGGCCAAGAGACTG	CAGAAGGAGGCCAGCATAAG
Jagged1	AACAAAGCTATCTGCCGACAGG	GGCTGATGAGTCCCACAGTAATTC
Jagged2	TTGGTGGCAAGAACTGCTCAGT	GCTGTCACAGATGCAGGAGAAGTT
Dll1	ACTGTACTCACCATAAGCCGTGCA	TCAGCTCACAGACCTTGCCATAGA
Dll4	CACTTGCCACGATCTGGAGAAT	TGCCCACAAAGCCATAAGGA
Hes1	GCACAGAAAGTCATCAAAGCC	TTGATCTGGGTCATGCAGTTG
Hes6	GCCGGATTTGGTGTCTACAT	TCCTGAGCTGTCTCCACCTT
HeyL	CAGATGCAAGCCCGGAAGAA	ACCAGAGGCATGGAGCATCT
Hey1	TGAATCCAGATGACCAGCTACTGT	TACTTTCAGACTCCGATCGCTTAC
β-actin	CAGGCATTGCTGACAGGATG	TGCTGATCCACATCTGCTGG

Supplemental Table 2. List of antibodies used for Western blot and Immunofluorescence.

Antibody	Source and Catalog no.	Analysis
Monoclonal mouse-anti-Pax7	DSHB # PAX7	WB/IF
Monoclonal mouse-anti-MyoD	Santa Cruz Biotechnology # 377460	WB/IF
Monoclonal mouse-anti-Myogenin	DSHB # F5D	WB/IF
Polyclonal TWEAK Receptor/Fn14	Cell Signaling Technology # 4403	WB
Monoclonal rabbit-anti-GAPDH	Cell Signaling Technology # 2118	WB
Monoclonal rabbit-anti-Cleaved Notch1	Cell Signaling Technology # 4147	WB
Polyclonal goat-anti-Notch1	Santa Cruz Biotechnology # 6014	WB
Polyclonal rabbit-anti-phospho-STAT2(Y690)	Cell Signaling Technology # 4441	WB
Monoclonal rabbit-anti-STAT2	Cell Signaling Technology # 72604	WB
Polyclonal rabbit-anti-phospho-STAT3 (S727)	Cell Signaling Technology # 9134	WB
Monoclonal rabbit-anti-phospho-STAT3 (Y705)	Cell Signaling Technology # 9145	WB
Monoclonal rabbit-anti-STAT3	Cell Signaling Technology # 30835	WB
Monoclonal rabbit-anti-phospho-STAT5 (Y694)	Cell Signaling Technology # 4322	WB
Monoclonal rabbit-anti-STAT5	Cell Signaling Technology # 94205	WB
Monoclonal mouse-anti-CD266 (ITEM-4-Fn14)	e-Bioscience # 14-9018-82	IF/Flow
Rat anti-mouse IgG2b FITC	Invitrogen # 11-4220-82	Flow
PE Rat Anti-mouse-anti- CD31	BD Pharmingen # 553373	Flow
Monoclonal Rat Anti-mouse-anti-CD45 PE	Invitrogen # 12-0451-82	Flow
Monoclonal Rat Anti-mouse-anti-Ly-6A (Sca-1) PE	e-Bioscience # 12-5981-82	Flow
Monoclonal Rat Anti-mouse-anti-Ter119 PE	e-Bioscience # 12-5921-82	Flow
Monoclonal mouse-anti-α7-integrin APC	Miltenyi Biotec # 130-102-717	Flow
Monoclonal mouse-anti-Myosin heavy chain (embryonic)	DSHB # F1.652	WB/IF
Polyclonal rabbit-anti-Laminin	Sigma # L9393	IF
Polyclonal rabbit-anti-Dystrophin	Abcam # 15277	IF
Goat anti-Mouse IgG1 Alexa Fluor 568	Life Technologies # A21124	IF
Goat anti-Mouse IgG2 Alexa Fluor 594	Life Technologies # A211135	IF
Goat anti-Rabbit IgG Alexa Fluor 488	Life Technologies # 11034	IF
Donkey anti-Rabbit IgG Alexa Fluor 555	Invitrogen # A31572	IF

Supplemental Table 3. Mean TPM values for WT myoblasts incubated in growth medium.

Gene name	Mean TPM values	Gene name	Mean TPM values
Ccng1	621.1946774	Lbh	249.6727904
Cxadr	35.79813652	Lif	2.59100748
Calcrl	12.72959725	Ncoa3	9.162479514
Cdk19	24.19957131	Notch1	22.85663415
Ccnd1	330.2630867	Notch2	37.60329254
Ccnd2	104.5287759	Pax3	4.398175455
Hgf	8.794593864	Six2	27.12187878
Gata6	1.084727413	Sox9	35.29842376
Fgfr2	0.948072161	Tead3	10.02624689
Apc2	1.818422341	Tgfbr3	1.548204592
Enpp1	0.989866001	Il6	3.397958934
Nkx2-5	0.326197706	Il15	0.419057697
Agtr1a	1.034336915	Cxcl10	17.12602464
Ccn3	4.104003524	Pdgfb	4.03449691
Cav2	8.514190025	Camk2a	5.321223919
Cdkl5	0.551536088	Camk2b	0.897912176
Ednra	3.094047633	Ifnb1	0
Cdkn2d	37.22444052	Ifna4	0
E2f4	60.58188387	Mmp2	164.1831704
Cdkn1a	587.4721457	Mmp9	0.485111336
Igfbp3	131.4441299	Aldh1a1	0.545122263
Csrp3	28.55942468	Birc5	72.57266062
Cxcl9	0.128365652	Socs2	61.92687082
Neu2	0.49052196	Socs3	17.30150569
Smyd1	66.58618927	Socs5	38.53755219
Lmod3	79.02987886	Sema3a	36.75054031
Myf6	14.00136893	Sema3e	95.10299035
Cav3	108.0811797	Fgf7	29.93319757
Myog	1319.010602	Vegfd	2.329139809
Rbm24	138.0515323	Il12a	7.134481129
Hopx	9.411419736	Mdm2	129.1157167
Fdps	31.01994176	Adamts1	17.27912129
Olfm2	0.642781463	Cx3cl1	16.3392645
Mef2c	18.38525838	Cxcl12	57.29570539
Ripor2	2.677810301	Dock4	1.020768725
Hif1an	25.38894483	Sirt1	19.41460565
Akap6	9.931814853	Adora2b	29.72144694
Dpf3	1.514276075	Aqp1	23.6756034
Fgfr1	78.48068766	Ackr3	93.70005894
Hmga2	122.8227459	Epha1	60.8916724
Jmjd1c	25.22217496	Epha4	1.221308232
		Nedd9	12.21753631
