# Supplementary material for: Fibroblast growth factor–inducible 14 regulates satellite cell self-renewal and expansion during skeletal muscle repair
Source: JCI Insight. 2025 Jan 28;10(5):e187825. doi: 10.1172/jci.insight.187825 (PMC11949035; doi:10.1172/jci.insight.187825)

UNEDITED GEL IMAGES

Full unedited gels for Figure 5G

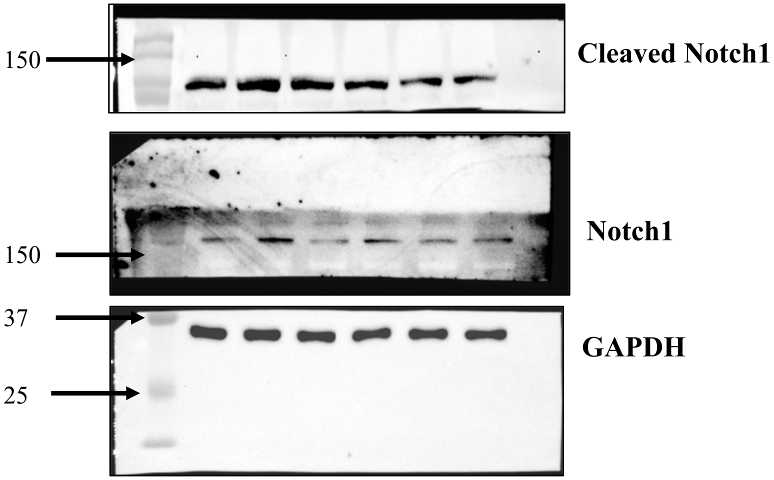

Full unedited gels for Figure 6B

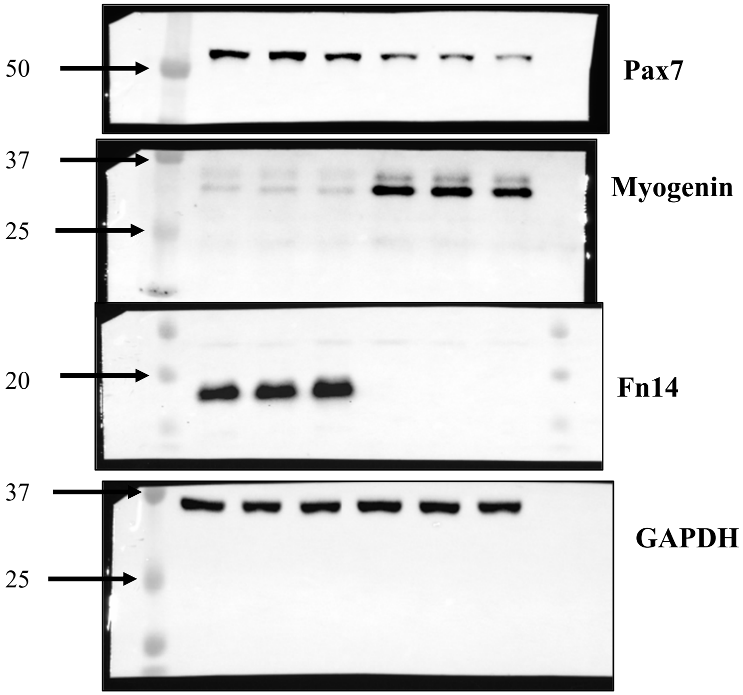

Full unedited gels for Figure 7B

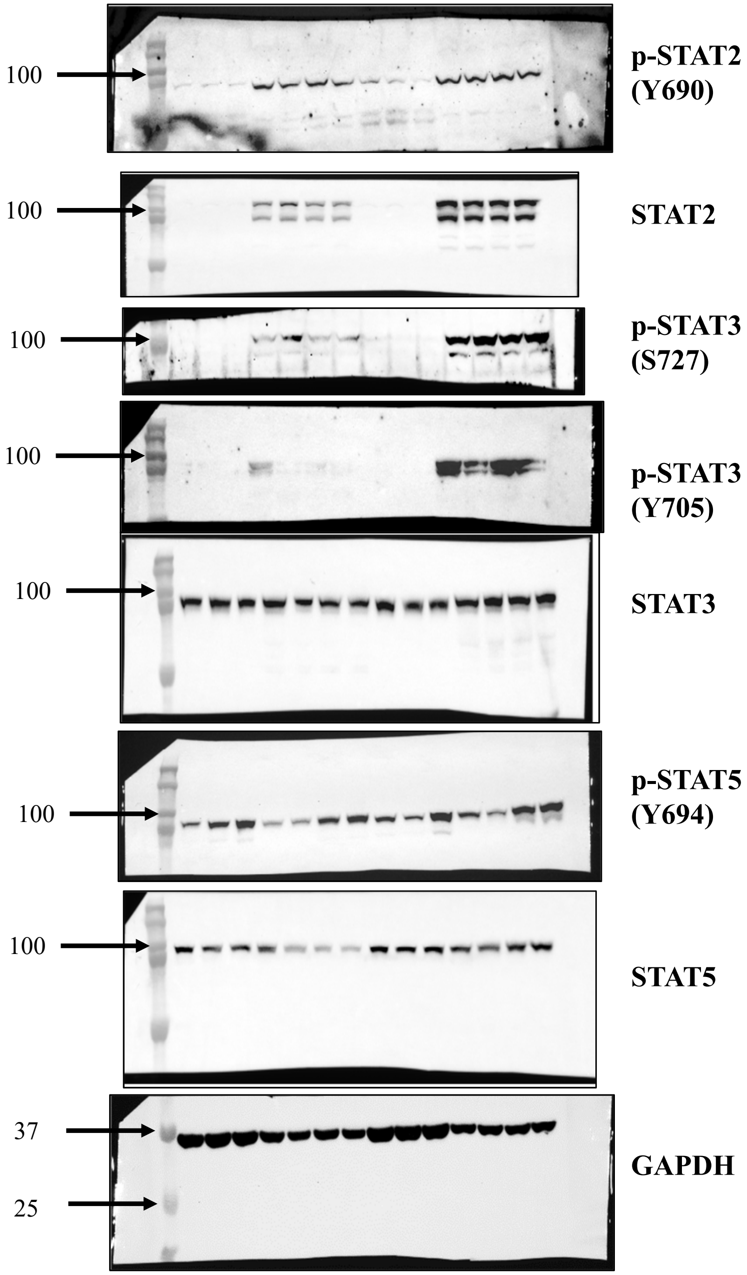

Full unedited gels for Figure 7C

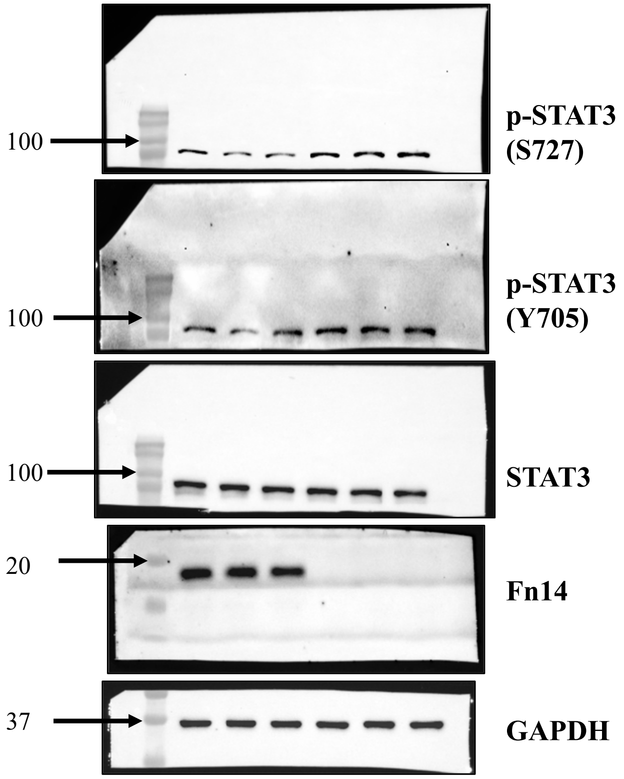

Full unedited gels for Figure 7F

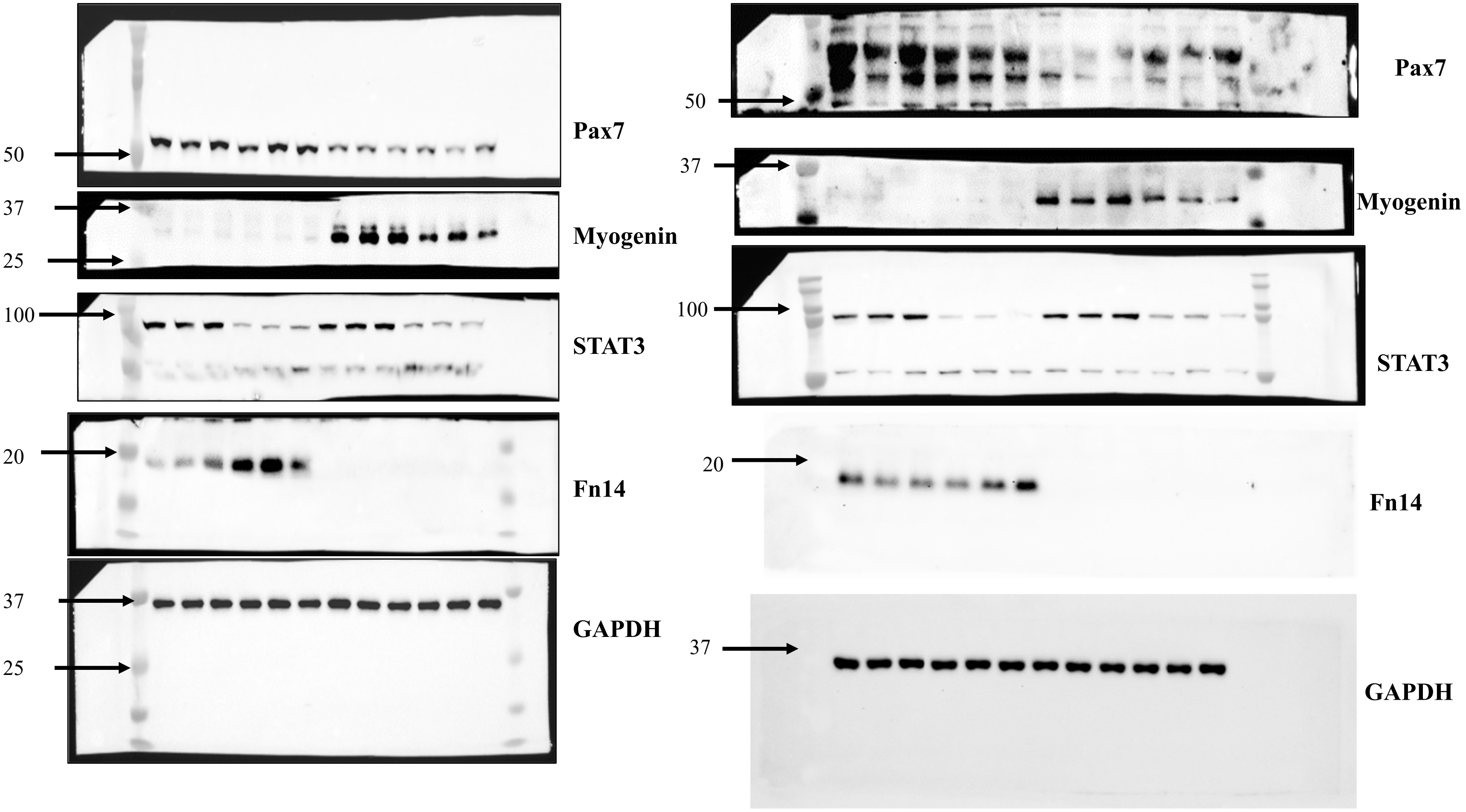

Full unedited gels for Figure 9A

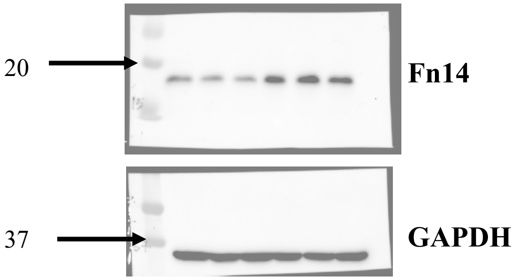

Supplement: Unedited blot and gel images [file jciinsight-10-187825-s137.pdf]
